# GABAA receptors in the nucleus raphe magnus modulate firing of neurons in the trigeminocervical complex

**DOI:** 10.1186/1129-2377-14-S1-P67

**Published:** 2013-02-21

**Authors:** W Supronsinchai, RJ Storer, J Hoffmann, AP Andreou, S Akerman, PJ Goadsby

**Affiliations:** 1 Headache Group, Department of Neurology, USA

## Background

Nociceptive transmission in the spinal cord is modulated by descending projections from the nucleus raphe magnus (NRM) receiving tonic inhibitory inputs from GABAergic neurons. The NRM modulates transmission of craniovascular nociception, which is related to head pain, in the trigeminocervical complex.

## Objective

To determine whether descending modulation of transmission of craniovascular nociception in the trigeminocervical complex involves GABA receptors in the NRM. If so, to characterize those GABA-receptors.

## Methods

We used a model of trigeminovascular nociception in Sprague Dawley rats that measures transmission of craniovascular nociception in the trigeminocervical complex (TCC) by the firing of TCC neurons evoked in response to electrical stimulation of afferents from the middle meningeal artery, its branches, and periarterial meninges (MMA). To determine whether GABA receptors in the NRM modulate this nociceptive transmission, and to characterize the modulation, we microinjected GABA, and GABAA- and GABAB-receptor agonists and antagonists into the NRM.

## Results

Microinjection of GABA into the NRM increased firing of TCC neurons evoked by stimulation of MMA afferents (p < 0.05). Moreover, microinjection of the GABAA receptor agonist, muscimol, into the NRM also increased evoked firing of TCC neurons. Whereas the GABAA receptor antagonist, bicuculline, decreased evoked firing of TCC neurons when microinjected into the NRM (p < 0.05). In contrast, microinjection of neither the GABAB receptor agonist, baclofen, or its antagonist, 2-hydroxysaclofen, into the NRM had no significant effect on the evoked firing of TCC neurons.

## Conclusion

This study shows that inhibition of NRM neurons by GABAA-receptor activation facilitates transmission of craniovascular nociception in the trigeminocervical complex. Our results suggest that GABAA receptors in the NRM play a role in the pathophysiology of migraine and other primary headache disorders.

